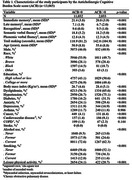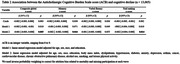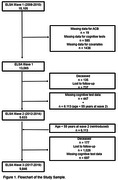# Anticholinergic burden and cognitive decline: findings from the ELSA‐Brasil cohort study

**DOI:** 10.1002/alz.089124

**Published:** 2025-01-09

**Authors:** Adriana Nancy Medeiros dos Santos, Natalia G Gonçalves, Alessandra C. Goulart, Maria Carmen Viana, Paulo A Lotufo, Isabela M Bensenor, Claudia Kimie Suemoto

**Affiliations:** ^1^ University of São Paulo Medical School, São Paulo Brazil; ^2^ University of São Paulo Medical School, São Paulo, SP Brazil; ^3^ University of São Paulo Medical School, SÃO PAULO Brazil; ^4^ University of São Paulo Medical School, São Paulo, São Paulo Brazil

## Abstract

**Background:**

Using multiple drugs with anticholinergic properties is common and might lead to cumulative anticholinergic toxicity and increased risk of cognitive decline. The cumulative adverse effects of anticholinergic medications that a person takes are called the “anticholinergic burden”. We hypothesized that a high anticholinergic burden may be a risk factor for cognitive decline. We aimed to investigate the longitudinal association between Anticholinergic Cognitive Burden (ACB) and cognitive decline in the ELSA‐Brasil study during eight years of follow‐up.

**Method:**

Participants were evaluated in three study waves (2008‐2010, 2012‐2014, and 2017‐2019). The ACB was calculated based on the medications in use. Cognitive performance was evaluated using a standardized battery of tests (immediate recall, late recall, recognition, semantic and phonemic verbal fluency, and the trail‐making tests). A global composite z‐score was derived from these tests. The association of ACB with cognitive performance over time was evaluated using linear mixed‐effects models, adjusted for sociodemographic and clinical variables.

**Result:**

A total of 15,105 participants were recruited and 2,040 were excluded, leaving 13,065 participants whose data were analyzed. Participants had a mean age of 51.7±9.0 years old, 55% were women, and 53% were white. During the follow‐up time, the ACB burden was associated with a decline in global cognition (β = ‐0.001, 95% CI = ‐0.013, ‐0.002, p = 0.004), memory performance (β = ‐0.002, 95% CI = ‐0.002, 0.000, p = 0.044) and executive function (β = ‐0.002, 95% CI = ‐0.004, 0.000, p = 0.014).

**Conclusion:**

During eight years of follow‐up, the ACB burden was associated with cognitive decline, particularly in the memory and executive function domains.